# Structural basis for CFTR inhibition by CFTR_inh_-172

**DOI:** 10.1073/pnas.2316675121

**Published:** 2024-02-29

**Authors:** Paul G. Young, Jesper Levring, Karol Fiedorczuk, Scott C. Blanchard, Jue Chen

**Affiliations:** ^a^Laboratory of Membrane Biology and Biophysics, The Rockefeller University, New York, NY 10065; ^b^Weill Cornell/Rockefeller/Sloan Kettering Tri-Institutional MD-PhD Program, New York, NY 10065; ^c^Department of Structural Biology, St. Jude Children’s Research Hospital, Memphis, TN 38101; ^d^HHMI, The Rockefeller University, New York, NY 10065

**Keywords:** CFTR, ion channel, inhibitor

## Abstract

The cystic fibrosis transmembrane conductance regulator (CFTR) is an anion channel that regulates electrolyte and fluid balance in epithelial tissues. While activation of CFTR is vital to treating cystic fibrosis, selective inhibition of CFTR is a potential therapeutic strategy for secretory diarrhea and autosomal dominant polycystic kidney disease. Although several CFTR inhibitors have been developed by high-throughput screening, their modes of action remain elusive. In this study, we determined the structure of CFTR in complex with the inhibitor CFTR_inh_-172 to an overall resolution of 2.7 Å by cryogenic electron microscopy. We observe that CFTR_inh_-172 binds inside the pore near transmembrane helix 8, a critical structural element that links adenosine triphosphate hydrolysis with channel gating. Binding of CFTR_inh_-172 stabilizes a conformation in which the chloride selectivity filter is collapsed, and the pore is blocked from the extracellular side of the membrane. Single-molecule fluorescence resonance energy transfer experiments indicate that CFTR_inh_-172 inhibits channel gating without compromising nucleotide-binding domain dimerization. Together, these data reconcile previous biophysical observations and provide a molecular basis for the activity of this widely used CFTR inhibitor.

## Significance

Cystic fibrosis transmembrane conductance regulator (CFTR) inhibitors, including CFTR_inh_-172, have been developed as therapeutic candidates to treat secretory diarrhea and autosomal dominant polycystic kidney disease. They are also widely used in laboratories to investigate the mechanisms underlying CFTR gating. This study offers a structural understanding of CFTR_inh_-172's mode of action, elucidating its ability to obstruct ion conduction and modulate channel gating. The molecular description of how CFTR_inh_-172 interacts with CFTR provides a structural foundation for its potency and efficacy. The observation that CFTR inhibitors and potentiators both interact with transmembrane helix 8 strengthens the notion that this helix serves as an allosteric link between the catalytic site and the channel gate and is therefore a hotspot for pharmacological modulation.

The cystic fibrosis transmembrane conductance regulator (CFTR) is an anion channel expressed in the apical membrane of epithelial cells (1, 2). Loss-of-function mutations in the *cftr* gene cause widespread salt and fluid dysregulation that leads to the autosomal recessive disease cystic fibrosis (1). By contrast, hyperactivation of CFTR is central to pathogenesis in secretory diarrhea and autosomal dominant polycystic kidney disease (ADPKD) (3–10). In both secretory diarrhea and ADPKD, cyclic adenosine monophosphate accumulation activates protein kinase A (PKA) (11–15). PKA-phosphorylation in turn activates CFTR (11, 12, 16–18), leading to excess fluid accumulation in the intestinal lumen and renal cysts, for secretory diarrhea and ADPKD, respectively (19, 20). CFTR hyperactivation has also been implicated in the pathogenesis of non-alcoholic steatohepatitis (21).

Despite functioning as an ion channel, CFTR belongs to the superfamily of ATP-binding cassette (ABC) transporters. It is composed of two transmembrane domains (TMDs) and two nucleotide-binding domains (NBDs) that are common to all ABC transporters, along with a cytosolic regulatory (R) domain specific to CFTR (22, 23). CFTR's activity is regulated at two levels. Phosphorylation by PKA releases the auto-inhibition imposed by the unphosphorylated R domain (24, 25). Once phosphorylated, adenosine triphosphate (ATP) binding promotes NBD dimerization and pore opening, whereas ATP hydrolysis leads to pore closure (26).

Substantial effort has been devoted to understanding CFTR in the context of cystic fibrosis. Disease-causing mutations have been extensively characterized, and small-molecule drugs that potentiate gating or correct folding of CFTR have been successfully developed for clinical use (27–38). By contrast, relatively few studies have addressed CFTR hyperactivation. Although secretory diarrhea is the second leading cause of death in children under 5 worldwide (20, 39, 40), and ADPKD is the most common inherited cause of end-stage renal disease (19), both conditions lack broadly effective, generalizable pharmacological treatments. Despite the therapeutic potential of CFTR inhibitors and although several small-molecule CFTR inhibitors have been identified (5, 6, 41, 42), their mechanisms and sites of action remain poorly understood.

In this study, we investigate the mechanism of CFTR_inh_-172, a highly efficacious CFTR inhibitor developed in the Verkman laboratory (5). This inhibitor was shown to block cholera toxin-induced intestinal fluid secretion (5) and suppress cyst growth in animal models of polycystic kidney disease (3). Substitution of pore-lining residues reduced the potency of CFTR_inh_-172, suggesting that it directly binds to CFTR (43). However, several studies have also suggested that CFTR_inh_-172 acts as a gating modulator rather than a classical pore-blocker (44, 45). Using cryogenic electron microscopy (cryo-EM), single-molecule fluorescence resonance energy transfer (smFRET), and electrophysiology, we have found that CFTR_inh_-172 binds within the pore, stabilizing the transmembrane helices in a nonconductive conformation without obstructing NBD dimerization. These findings enable us to propose a mechanism for CFTR_inh_-172 that reconciles previous observations.

## Results

### CFTR_inh_-172 Inhibits Wild-Type (WT) CFTR and the “Locked-Open” CFTR (E1371Q).

**Fig. 1.**
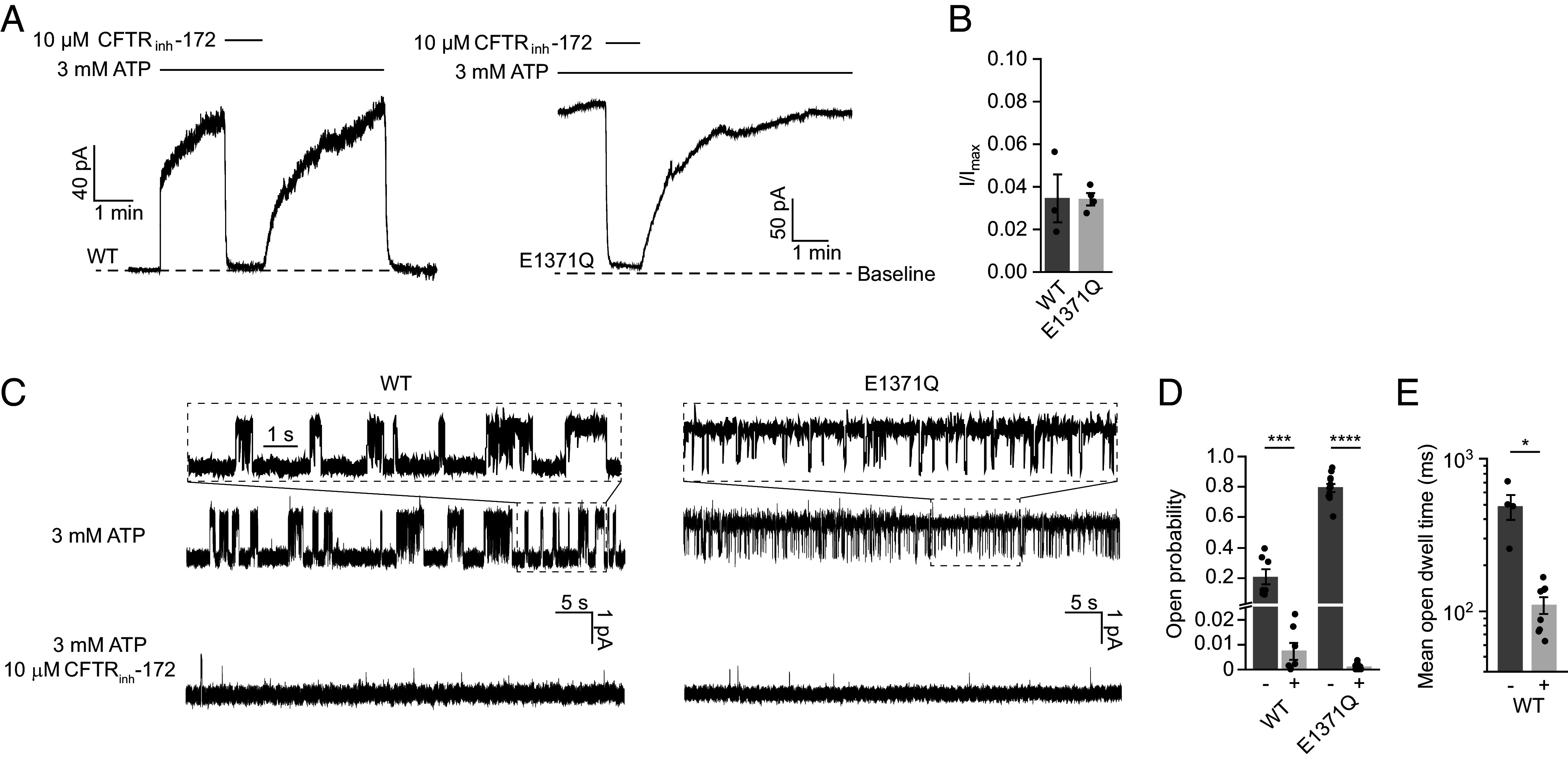
CFTR_inh_-172 inhibits WT CFTR and CFTR (E1371Q). (*A*) Example macroscopic current traces showing inhibition of WT CFTR and CFTR (E1371Q) by CFTR_inh_-172 in inside-out excised patches. CFTR was fully phosphorylated by PKA in the presence of 3 mM ATP before the displayed recordings. (*B*) Currents with 10 µM CFTR_inh_-172 (I) relative to currents without CFTR_inh_-172 (I_max_). Data represent means and standard error (SE) of 3 (WT) or 4 (E1371Q) patches. Individual data points are displayed as dots. (*C*) Example single-channel recordings of PKA-phosphorylated WT CFTR and CFTR (E1371Q) reconstituted in synthetic lipid bilayers with and without CFTR_inh_-172. Recordings were made at −150 mV. Upward deflections correspond to opening. (*D*) Open probabilities of WT CFTR and CFTR (E1371Q) with and without CFTR_inh_-172. Data represent means and SE of 7 (WT, with or without CFTR_inh_-172), 11 (E1371Q, without CFTR_inh_-172), or 9 (E1371Q, with CFTR_inh_-172) bilayers. Statistical significance was tested by one-way ANOVA (***P = 2 × 10^−4^, ****P < 10^−10^). (*E*) Mean open dwell time of WT CFTR with and without CFTR_inh_-172. Data represent means and SE of 4 (without CFTR_inh_-172) or 8 (with CFTR_inh_-172) bilayers. Statistical significance was tested using two-tailed Student’s *t* test (*P = 0.025).

Previously, the hydrolysis-deficient CFTR (E1371Q) variant has been used to obtain high-resolution structures of CFTR in complex with modulators, leveraging its ability to stabilize the canonical NBD dimer (25, 36, 37, 46, 47). To test whether the same variant is suitable to study CFTR_inh_-172, we characterized the effects of CFTR_inh_-172 on both WT CFTR and CFTR (E1371Q) (Fig. 1). In excised inside-out membrane patches (Fig. 1 *A* and *B*), application of 10 µM CFTR_inh_-172 reduced the macroscopic current of WT CFTR by 96%. A comparable level of inhibition was observed with CFTR (E1371Q), despite its open dwell time being 1,000-fold longer than that of WT CFTR (48). Our data are consistent with previous work on WT CFTR and two other hydrolysis-deficient variants, D1370N and E1371S (45).

Next, we purified CFTR and reconstituted it into a synthetic planar lipid bilayer. Upon activation by PKA, single-channel currents were measured in the presence of 3 mM ATP with or without 10 µM CFTR_inh_-172 (Fig. 1*C*). CFTR_inh_-172 reduced the open probability of WT CFTR from 0.21 ± 0.05 (mean and SE) to 0.007 ± 0.003, whereas that of CFTR (E1371Q) decreased from 0.79 ± 0.03 to 0.0011 ± 0.0004 (Fig. 1*D*). Previous studies had reported conflicting evidence regarding the effect of CFTR_inh_-172 on open dwell time (44, 45). Here, we observed both a large effect on closed dwell time and a fivefold reduction in mean open dwell time, from 487 ± 92 ms to 109 ± 14 ms for WT CFTR (Fig. 1*E*), consistent with findings from Kopeikin and colleagues (45).

Taken together, both macroscopic and single-channel current measurements indicate that CFTR_inh_-172 inhibits CFTR (E1371Q) as effectively as it does WT CFTR, suggesting a comparable mechanism of inhibition.

### CFTR_inh_-172 Binds within the Pore.

**Fig. 2.**
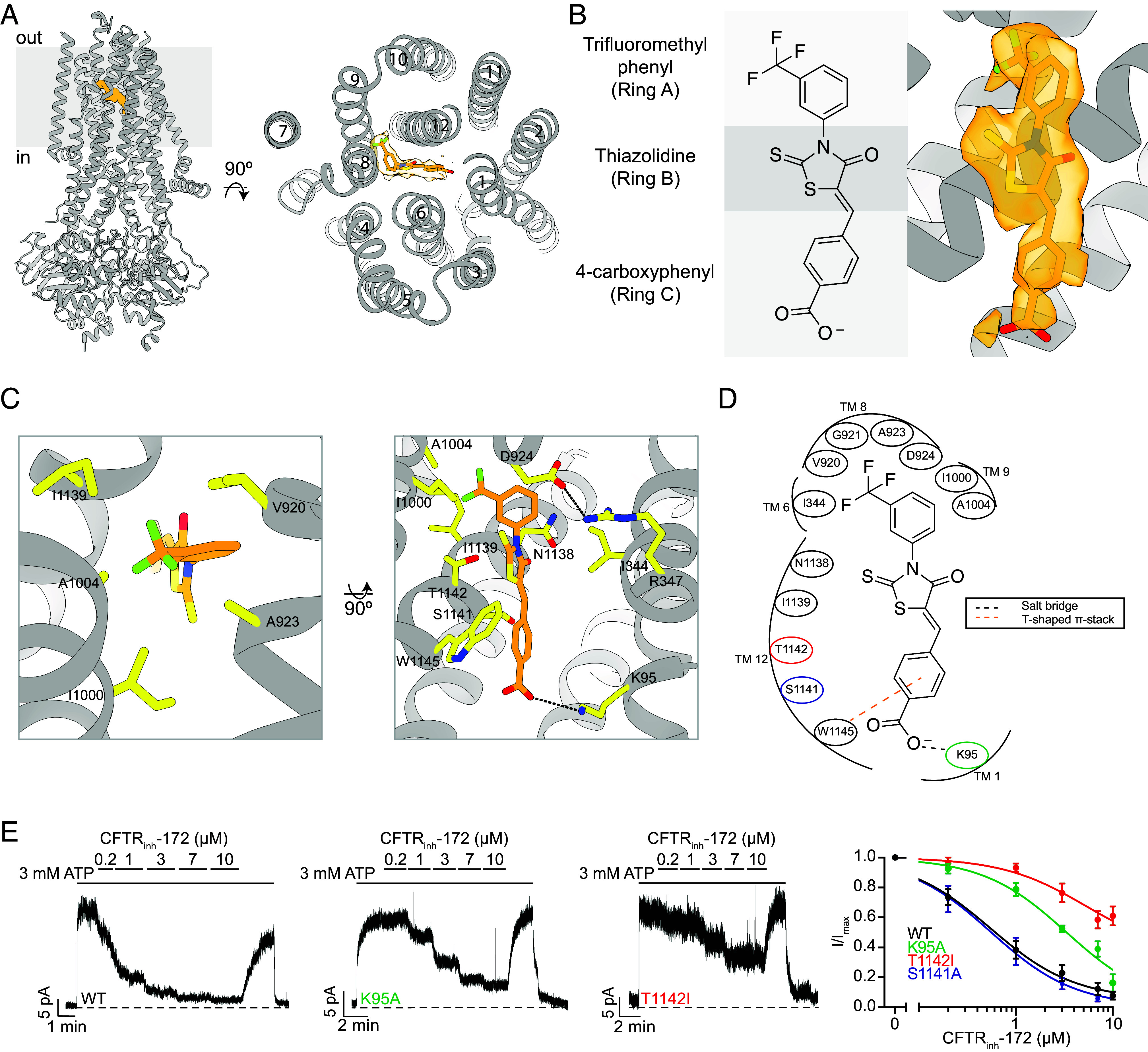
CFTR_inh_-172 makes specific interactions with the CFTR inner vestibule. (*A*, *Left*) Structure of the CFTR (E1371Q)/CFTR_inh_-172 complex. (*Right*) A view of the structure looking down the long axis of the CFTR pore with CFTR_inh_-172 modeled as orange sticks surrounded by cryo-EM density. (*B*, *Left*) Structure of CFTR_inh_-172 with the three functional rings delineated by gray rectangles. (*Right*) Structure of CFTR_inh_-172 modeled into its binding site within the inner vestibule surrounded by cryo-EM density. (*C*) Interacting residues within 4.5 Å of CFTR_inh_-172. (*Left*) The hydrophobic pocket of the CFTR_inh_-172 binding site. (*Right*) The solvent-exposed pocket of the CFTR_inh_-172 binding site. CFTR_inh_-172/K95 and R347/D924 salt bridges are shown as black dashed lines. (*D*) Schematic drawing of CFTR-inhibitor interactions. All residues within 4.5 Å of the inhibitor are depicted. Residues substituted in inside-out path-clamp electrophysiology are indicated with colored circles. (*E*) Example macroscopic current traces showing titration of CFTR_inh_-172 onto WT, K95A, or T1142I CFTR in inside-out excised patches. CFTR was fully phosphorylated by PKA in the presence of 3 mM ATP before CFTR_inh_-172 titration. (*Right*) Dose–response curves for CFTR_inh_-172 binding site variants. The mean current in the presence of 3 mM ATP alone was used to normalize current elicited at each CFTR_inh_-172 concentration. Dose–response curves were fitted with the Hill equation to estimate *IC_50_* values for each variant. Hill coefficients were fixed to 1. Each data point represents mean and SE determined from three to five patches.

To identify the binding site of CFTR_inh_-172, we determined the cryo-EM structure of inhibitor-bound CFTR using phosphorylated CFTR (E1371Q) (Fig. 2 and *SI Appendix*, Fig. S1). The structure was determined to an overall resolution of 2.7 Å. In the presence of CFTR_inh_-172 and ATP, CFTR (E1371Q) adopts an NBD-dimerized, pore-closed conformation distinct from any previously observed structures (Fig. 2*A*). The density for CFTR_inh_-172, as strong as the protein main chain atoms, was observed inside the pore, at a position corresponding to the membrane outer leaflet (Fig. 2*A*). The chemical structure of CFTR_inh_-172 can be divided into three rings (Fig. 2*B*): a central thiazolidine ring (ring B) with a (3-trifluoromethyl)phenyl substitution at position 3 (ring A) and a (4-carboxyphenyl)methylene substitution at position 5 (ring C). The density shows well-defined features corresponding to the trifluoromethyl phenyl and the heavy sulfur atoms in the thiazolidine ring (Fig. 2*B*). The density for ring C is not as well-defined, indicating that this moiety may be mobile.

CFTR_inh_-172 binds within the CFTR pore and interacts with both TMDs (Fig. 2*A*). The three rings of the compound form an elongated shape wedged between transmembrane helices (TMs) 1, 6, 8, 9, and 12. Whereas ring A and ring B are completely buried, ring C is exposed to the aqueous ion-conduction pathway (Fig. 2*A*). With this structure, we can now better understand previous structure–activity-relationship (SAR) analyses (5, 49). Specifically, these studies showed that the trifluoromethyl group (CF_3_) is essential for inhibition and that the addition of polar substituents or removal of CF_3_ on ring A diminished activity (49). The highest potency is achieved when CF_3_ is at position 3 of ring A, as in CFTR_inh_-172 (5, 49). In the structure, the trifluoromethyl group fits snugly into a hydrophobic cavity, establishing van der Waals interactions with five hydrophobic residues on TMs 6, 8, 9, and 12 (Fig. 2 *C* and *D*). If the CF_3_ substitution were at positions 2 or 4, many of these interactions would be lost, resulting in lower inhibitory potency, as observed (49). The SAR studies further demonstrate the significance of negative or polar substitutions on ring C (49). Indeed, we observe that this ring is positioned within a spacious, solvent-exposed cavity surrounded by numerous charged and polar residues, including K95 on TM1 and N1138, S1141, and T1142 on TM12 (Fig. 2 *C* and *D*). The carboxy group on CFTR_inh_-172 forms a salt bridge with K95 (Fig. 2 *C* and *D*). Consistent with this observation, esterification or amidation of the carboxy group in CFTR_inh_-172 resulted in inactive compounds (49), presumably due to the loss of this interaction. The reciprocal change on CFTR, substitution of K95 with alanine, resulted in a nearly sevenfold decrease in potency, increasing the half maximal inhibitory concentration (*IC_50_*) of CFTR_inh_-172 from 0.6 ± 0.1 µM to 3.5 ± 0.9 µM (Fig. 2*E*). Perturbation of the binding site by T1142I substitution increased the *IC_50_* to 5.3 ± 3.1 µM (Fig. 2*E*). The effect of the T1142I substitution is most likely due to steric hindrance, as the side chain of T1142 is close to the methylene group at position 2 of ring B (Fig. 2 *C* and *D*). The reciprocal modification on the inhibitor, adding a methyl at this position, was shown to increase the *IC_50_* to 8 µM (49). By contrast, alanine substitution of a non-interacting residue, S1141, had no effect on inhibitory potency (Fig. 2*E* and *SI Appendix*, Fig. S2).

The structure also offers a molecular explanation for previous data showing that substitution of R347 with alanine decreases the potency of CFTR_inh_-172 by over 30-fold and that R347D substitution nearly eliminated its inhibitory effect (43). Although R347 does not interact with the inhibitor directly, it forms a salt bridge with D924, creating a surface against which the inhibitor is tightly packed (Fig. 2*C*). Substitutions at position 347 are likely to modify the structure of the binding site, consequently reducing the inhibitory activity.

### CFTR_inh_-172 Stabilizes a Closed Conformation of CFTR.

**Fig. 3.**
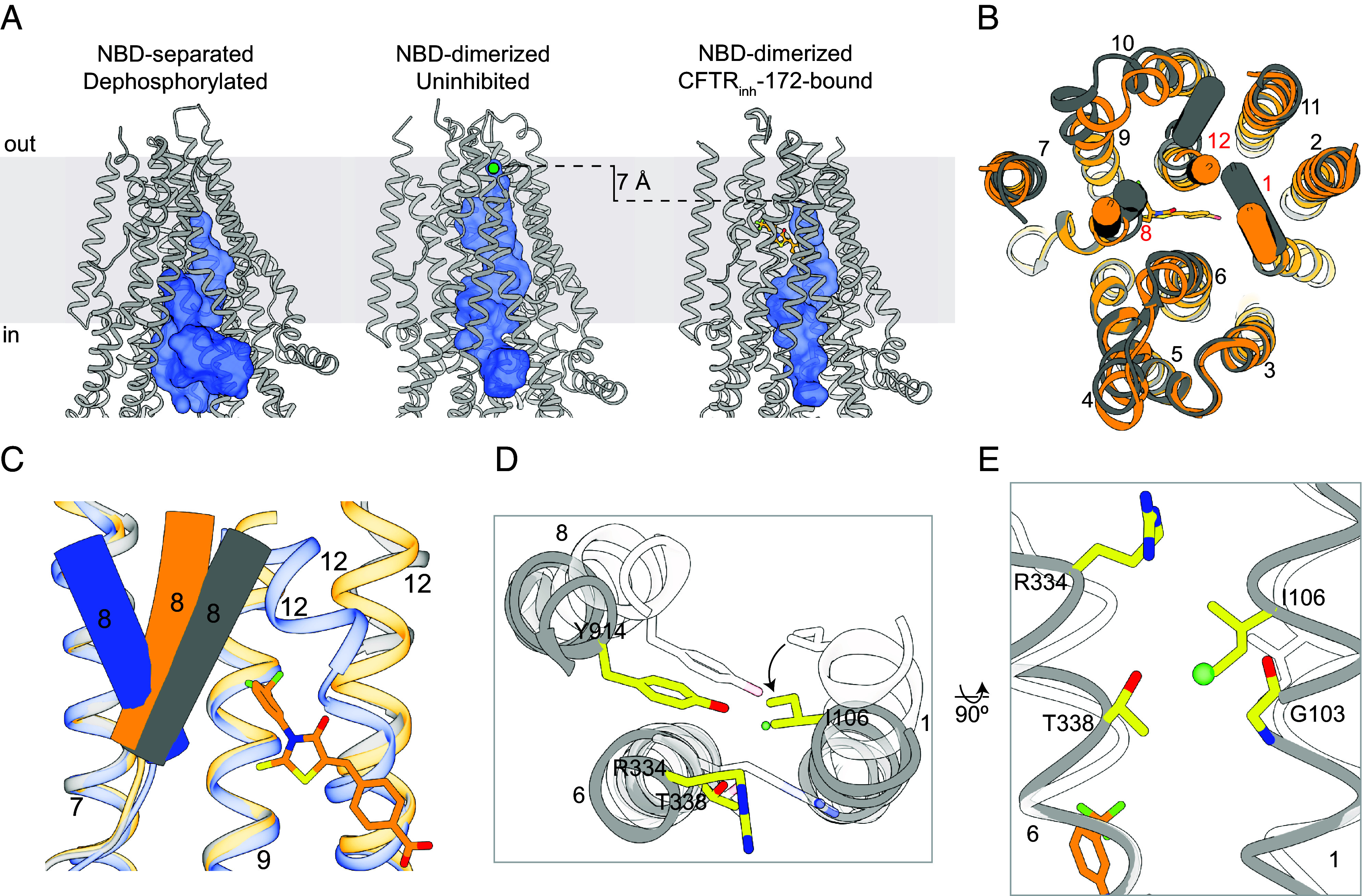
CFTR_inh_-172 stabilizes a closed conformation of CFTR. (*A*) Comparison of the pore (shown as a blue surface) in NBD-separated, dephosphorylated (PDB 5UAK), NBD-dimerized, uninhibited (PDB 7SVD), and NBD-dimerized, CFTR_inh_-172-bound CFTR, as defined by a spherical probe with a radius of 1.7 Å. Chloride is modeled as a green sphere in the NBD-dimerized, uninhibited structure. CFTR_inh_-172 is shown as an orange ball-and-stick model. (*B*) Overlay of NBD-dimerized, CFTR_inh_-172-bound CFTR (orange) with NBD-dimerized, uninhibited CFTR (gray). TMs 1, 8, and 12 are shown as cylinders. (*C*) Local conformational comparison of NBD-dimerized, CFTR_inh_-172-bound (orange), NBD-dimerized, uninhibited (gray), and NBD-separated, dephosphorylated (blue) CFTR. This figure was obtained by superposition of NBD2 and TMs 5, 7, 8, 9, and 12 from the three conformations. Individual TMs are labeled. (*D*) Extracellular view of local superposition of helices and residues comprising the selectivity filter in NBD-dimerized, CFTR_inh_-172-bound (gray/yellow) and NBD-dimerized, uninhibited (transparent white) CFTR. The chloride ion observed in the NBD-dimerized, uninhibited structure is shown as a green sphere. (*E*) Local superposition of TMs 1 and 6 in NBD-dimerized, CFTR_inh_-172-bound (gray/yellow) and NBD-dimerized, uninhibited (transparent white) CFTR. The chloride ion observed in the NBD-dimerized, uninhibited structure is shown as a green sphere.

Previous structural studies of human CFTR have revealed the conformational changes required for pore opening (Fig. 3*A*). Dephosphorylated CFTR exhibits an NBD-separated conformation with an inner vestibule open to the cytosol but the pore closed off to the extracellular space (Fig. 3*A*) (50). In the presence of ATP, phosphorylated CFTR (E1371Q) forms an NBD-dimerized conformation (49), in which the pore is open and a dehydrated chloride ion is bound at the selectivity filter near the extracellular entrance (51) (see accompanying paper) (Fig. 3*A*). A comparison of these two structures reveals that phosphorylation and ATP binding cause the NBDs and TMDs to move toward the central axis essentially as rigid bodies. However, local conformational changes of TMs 8 and 12 are also critical for CFTR gating (25).

In the presence of CFTR_inh_-172, CFTR adopts a conformation distinct from either structure (Fig. 3*A*). The NBDs form a dimer similar to that observed in the uninhibited structures of CFTR (E1371Q) (25, 46). The TMDs undergo global rigid-body movements toward each other, but TMs 1, 8, and 12 are positioned differently (Fig. 3*B*). Local structural superposition shows that the extracellular segment of TM8 is stabilized in a conformation intermediate between the NBD-separated and -dimerized conformations (Fig. 3*C*). Furthermore, the anion selectivity filter (51) (see accompanying paper) collapses, as TM1 undergoes a ~5° rotation that places the side chain of I106 at the position occupied by the chloride ion in the uninhibited structure (Fig. 3 *D* and *E*). This repositioning of the TMs also leads to a complete closure of the lateral exit between TMs 1 and 6 that connects the selectivity filter to the extracellular space (Fig. 3*E*).

### CFTR_inh_-172 Allosterically Inhibits ATP Turnover.

**Fig. 4.**
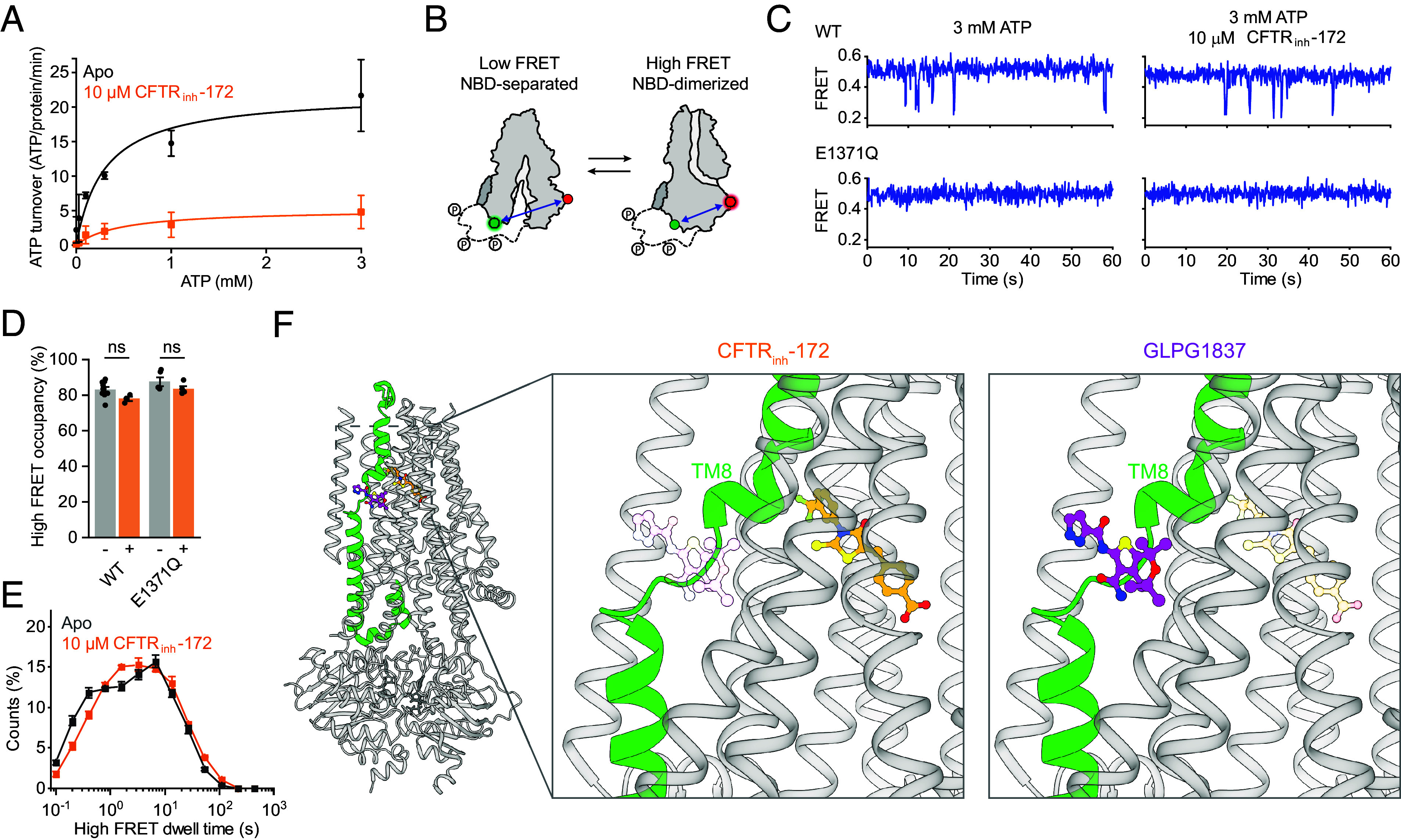
Allosteric inhibition of ATP-dependent gating. (*A*) Effect of CFTR_inh_-172 on steady-state ATP hydrolysis by PKA-phosphorylated WT CFTR. Data represent means and SE for 3 (without CFTR_inh_-172) or 4 (with CFTR_inh_-172) measurements and are fitted with the Michaelis–Menten equation. Without CFTR_inh_-172, the *K_m_* for ATP was 0.3 ± 0.1 mM and *k_cat_* was 22 ± 3 ATP/protein/min. With CFTR_inh_-172, the *K_m_* was 0.47 ± 0.29 mM and *k_cat_* was 5.2 ± 1.0 ATP/protein/min. (*B*) Schematic of individual CFTR molecules labeled for smFRET imaging. Green and red circles indicate fluorophore positions. (*C*) Example smFRET traces for PKA-phosphorylated WT CFTR and CFTR (E1371Q) with and without CFTR_inh_-172. (*D*) Effects of CFTR_inh_-172 on probabilities of NBD dimerization for WT CFTR and CFTR (E1371Q). Data represent means and SE for 9 (WT, without CFTR_inh_-172), 3 (WT, with CFTR_inh_-172), 5 (E1371Q, without CFTR_inh_-172), or 4 (E1371Q, with CFTR_inh_-172) measurements. Statistical significance was tested by one-way ANOVA (ns, not significant). (*E*) Dwell-time distributions for NBD dimerization for WT CFTR with and without 10 µM CFTR_inh_-172. Data represent means and SE for 8 (without CFTR_inh_-172) or 3 (with CFTR_inh_-172) measurements. (*F*) Cartoon representation of CFTR bound to CFTR_inh_-172 (*Left*). TM 8 is colored green. The potentiator GLPG1837 (colored magenta) was overlayed onto the CFTR_inh_-172-bound structure. Closeup views of the CFTR_inh_-172 (*Middle*) and GLPG1837 (*Right*) binding sites. In each view, the other compound is shown as a transparent ball-and-stick model to emphasize their relative positions.

Our structural analysis clearly shows that binding of CFTR_inh_-172 leads to pore closure by trapping the TM helices in a non-conductive state (Fig. 3). As CFTR gating is coupled to ATP hydrolysis, we tested whether CFTR_inh_-172 changes the ATP turnover rate (Fig. 4*A*). We found that the presence of 10 µM CFTR_inh_-172 decreased saturating ATP turnover (*k_cat_*) by approximately fourfold, from 22.0 ± 2.2 to 5.2 ± 1.0 ATP/protein/min (Fig. 4*A*). The Michaelis–Menten constant (*K_m_*) for ATP was not significantly changed (Fig. 4*A*).

To assess the mechanism by which ATP hydrolysis is inhibited by CFTR_inh_-172, we used a recently established smFRET assay, which reports on the conformational state of CFTR’s NBDs (38). In this assay, position 388 in NBD1 and position 1435 in NBD2 were labeled with donor and acceptor fluorophores (Fig. 4*B*). Conformational isomerizations of individual CFTR molecules were monitored as transitions between a low FRET efficiency (0.25 ± 0.01) NBD-separated state and a high FRET efficiency (0.49 ± 0.02) NBD-dimerized state. As we have previously reported (38), at a saturating (3 mM) ATP concentration, WT CFTR predominantly occupies NBD-dimerized conformations with brief excursions to the NBD-separated state (Fig. 4*C*). The presence of 10 µM CFTR_inh_-172 did not significantly affect the probability of NBD dimerization or the dwell time of the NBD-dimerized state for WT CFTR (Fig. 4 *D* and *E*). CFTR_inh_-172 also did not affect the conformational dynamics of CFTR (E1371Q), which remained predominantly NBD-dimerized (Fig. 4 *C*–*E*). These data indicate that CFTR_inh_-172 does not prevent NBD dimerization but rather slows progression through the gating cycle while the NBDs are dimerized.

These observations lead us to consider a possible mechanism for inhibition of ATP hydrolysis. Our recent study showed that conformational changes within NBD-dimerized CFTR governed by ATP turnover are required for chloride conductance (38). Potentiators Ivacaftor and GLPG1837 enhance channel activity by increasing pore opening while the NBDs are dimerized. Additionally, the potentiators increase ATP turnover (38). In comparing the structure of CFTR (E1371Q) bound to GLPG1837 with that bound to CFTR_inh_-172, we observe that the CFTR_inh_-172 site is located along the pore-lining side of the TM8 hinge region, in direct juxtaposition to the potentiator binding site (Fig. 4*F*). As TM8 links ATP hydrolysis and pore opening (36, 52), we propose that CFTR_inh_-172 inhibits ATP turnover via an allosteric mechanism involving TM8, similar in nature but opposite in effect to that of the potentiators. However, the exact step of the ATP hydrolysis cycle perturbed by CFTR_inh_-172 remains unclear. It is possible that CFTR_inh_-172 reduces the hydrolysis rate by slowing post-hydrolytic nucleotide exchange or promoting occupancy of a state that is not transited by WT CFTR during physiological gating.

## Discussion

Typically, ion channel inhibitors are classified into two categories: pore blockers that bind within the ion conduction pathway to occlude the pore and gating inhibitors that impair channel opening by stabilizing the closed state (53). However, CFTR_inh_-172 presents a perplexing case, as it has been shown to interact with residues within the pore while also impairing gating (43, 44). Through cryo-EM and smFRET analyses, we now have a structural understanding of CFTR_inh_-172 inhibition that reconciles earlier findings. The binding site of CFTR_inh_-172 is located within the pore, nestled in a cavity lined by R347, a residue whose substitution significantly reduces the potency of CFTR_inh_-172 (43). Local conformational changes of TMs 1, 8, and 12 in the CFTR_inh_-172-bound structure cause a collapse of the chloride selectivity filter and the extracellular exit. Based on kinetic analysis, Hwang and colleagues had predicted that CFTR_inh_-172 induces conformational change in CFTR (45). The nature of this change is now revealed at a molecular level. Additionally, we found that CFTR_inh_-172 inhibits ATP hydrolysis through an allosteric mechanism that we hypothesize is similar to that of potentiators Ivacaftor and GLPG1837 but with an opposite functional effect. These observations corroborate the hypothesis that conformational shifts in TM8 link ATP hydrolysis at the NBDs with the state of the pore.

Electrophysiological measurements in our laboratory (Fig. 1) and in other studies (45) demonstrate that CFTR_inh_-172 inhibits WT CFTR and several hydrolysis-deficient variants to similar extents. Although it is theoretically possible that this molecule inhibits WT CFTR and each variant through different mechanisms, it is more likely that the mechanism of action is similar. Indeed, the structure determined with CFTR (E1371Q) is entirely consistent with earlier SAR studies (5, 49). Substitutions at the structurally identified binding site made in the WT CFTR background reduced the potency of CFTR_inh_-172 (43) (Fig. 2*E*). Additionally, smFRET studies reveal that CFTR_inh_-172 does not affect NBD isomerization in WT CFTR or CFTR (E1371Q) (Fig. 4 *C* and *D*). These data strongly suggest that the mode of action revealed in this study represents a general mechanism for CFTR_inh_-172. However, as E1371Q substitution stabilizes the NBDs in a canonical dimerized conformation, it is possible that CFTR_inh_-172 induces local changes at the ATPase site that are obscured in our structure. Further studies will be pursued to identify structural re-arrangements of the WT channel within the NBD-dimerized state, as these play a key role in coupling ATP hydrolysis to channel gating.

Finally, the congruence between structural and functional data not only offers intellectual satisfaction but also opens up new avenues for enhancing the potency and specificity of CFTR_inh_-172. Specifically, CFTR interacts with ring C of CFTR_inh_-172 primarily through the K95 salt-bridge and an edge-to-face π-stacking interaction with W1145. It is possible that analogs of CFTR_inh_-172, with modifications on ring C that establish additional interactions with nearby polar residues on CFTR will have enhanced potency and specificity.

## Methods

### Cell Culture.

Sf9 cells (Gibco, catalog number 11496015, lot number 1670337) were grown at 27 °C in Sf-900 II SFM medium (Gibco) supplemented with 5% (v/v) fetal bovine serum (FBS) and 1% (v/v) antibiotic-antimycotic (Gibco). HEK293S GnTI^−^ cells (American type culture collection (ATCC) CRL-3022, lot number 62430067) were cultured at 37 °C in Freestyle 293 medium (Gibco) supplemented with 2% (v/v) FBS and 1% (v/v) antibiotic-antimycotic. CHO (Chinese hamster ovary)-K1 cells (ATCC CCL-61, lot number 70014310) were cultured at 37 °C in Dulbecco’s Modified Eagle Medium/Nutrient Mixture F-12 (DMEM/F-12) (ATCC) supplemented with 10% (v/v) FBS and 1% (v/v) GlutaMAX (Gibco).

### Mutagenesis.

CFTR variants were generated using the SPRINP mutagenesis method (*SI Appendix*, Table S2) (54). Briefly, mutagenic primers were designed to be complementary to the template plasmid except for the mutated bases and to be 15 to 45 nucleotides in length. Plasmid containing CFTR cDNA was amplified in separate reactions containing forward or reverse primer. The single-primer products of these reactions were combined and denatured at 95 °C for 5 min and gradually cooled to 37 °C over the next 5 min. The sample was then digested by DpnI for 4 h. Then, 5 µL of sample was added to 50 µL of competent XL2Blue cells for transformation and incubated on ice for 30 min. The bacteria were then heat-shocked at 42 °C for 45 s and allowed to recover on ice for 2 min. Following that, 200 µL of warmed SOC media (Invitrogen) was then added directly to the cells, and the mixture was allowed to shake at 225 RPM in a 37 °C incubator for 30 min. Then, 200 µL of this mixture was spread on LB/ampicillin plates and left to incubate at 37 °C overnight. Random colonies were then picked and expanded in LB/ampicillin. Plasmid DNA was then purified (QIAGEN Plasmid Kit) and sequenced (Genewiz).

### Patch-Clamp Electrophysiology.

CHO (ATCC CCL-61, lot number 70014310) cells were maintained in DMEM/F12 (ATCC) supplemented with 10% (v/v) heat-inactivated FBS and 1% GlutaMAX (Gibco) at 37 °C. The cells were seeded in 35-mm cell culture dishes (Falcon) twenty-four hours before transfection. Cells were transiently transfected with BacMam vector encoding C-terminally green fluorescent protein (GFP)-fused CFTR, using Lipofectamine 3000 (Invitrogen). Twelve hours after transfection, the medium was exchanged for DMEM/F12 supplemented with 2% (v/v) FBS and 1% (v/v) GlutaMAX, and incubation temperature was reduced to 30 °C. Patch-clamp recording was carried out after an additional 24 h.

The bath solution was 145 mM NaCl, 2 mM MgCl_2_, 5 mM KCl, 1 mM CaCl_2_, 5 mM glucose, 5 mM (4-(2-hydroxyethyl)-1-piperazineethanesulfonic acid (HEPES), and 20 mM sucrose (pH 7.4 with NaOH). Pipette solution was 140 mM NMDG, 5 mM CaCl_2_, 2 mM MgCl_2_, and 10 mM HEPES (pH 7.4 with HCl). Perfusion solution was 150 mM NMDG, 2 mM MgCl_2_, 1 mM CaCl_2_, 10 mM (ethylene glycol-bis(β-aminoethyl ether)-N,N,N',N'-tetraacetic acid (EGTA), and 8 mM Tris (pH 7.4 with HCl).

Recordings were carried out using the inside-out patch configuration with local perfusion at the patch. Recording pipettes were pulled from borosilicate glass (outer diameter 1.5 mm, inner diameter 0.86 mm, Sutter) to 1.5 to 3.0 MΩ resistance. Currents were recorded at 25 °C using an Axopatch 200B amplifier, a Digidata 1550 digitizer, and the pClamp software suite (Molecular Devices). Membrane potential was clamped at −30 mV. Current traces reflect inward currents with inverted signatures. Recordings were low-pass filtered at 1 kHz and digitized at 20 kHz.

For all measurements, CFTR was activated by exposure to PKA (Sigma-Aldrich) and 3 mM ATP. Displayed recordings were low-pass filtered at 100 Hz. Data were analyzed using Clampfit, GraphPad Prism, and OriginPro.

### Protein Expression and Purification.

CFTR constructs were expressed and purified as previously described (55, 56). Bacmids encoding human CFTR fused to a C-terminal PreScission Protease-cleavable GFP tag were generated in *Escherichia coli* DH10Bac cells (Invitrogen). Recombinant baculovirus was produced and amplified in Sf9 cells. HEK293S GnTl^−^ suspension cells, at a density of 2.0 to 3.0 × 10^6^ cells/mL, were infected with 10% (v/v) P3 or P4 baculovirus. Protein expression was induced by addition of 10 mM (final concentration) sodium butyrate 12 h after infection. The cells were cultured at 30 °C for an additional 48 h and then harvested by centrifugation.

Protein samples for cryo-EM were purified as follows. Cells were solubilized for 75 min at 4 °C in extraction buffer containing 1 to 1.25% (w/v) 2,2-didecylpropane-1,3-bis-b-D-maltopyranoside, 0.25% (w/v) cholesteryl hemisuccinate, 200 mM NaCl, 20 mM HEPES (pH 7.5 with NaOH), 2mM MgCl_2_, 10 μM dithiothreitol (DTT), 20% (v/v) glycerol, 2 mM ATP, 1 μg mL^−1^ pepstatin A, 1 μg mL^−1^ aprotinin, 100 μg mL^−1^ soy trypsin inhibitor, 1 mM benzamidine, 1 mM phenylmethylsulfonyl fluoride, and 3 μg mL^−1^ DNase I. Lysate was clarified by centrifugation at 75,000 g for 45 min at 4 °C and mixed with NHS-activated Sepharose 4 Fast Flow resin (GE Healthcare) conjugated with GFP nanobody, which had been pre-equilibrated in extraction buffer. After 2 h, the resin was packed into a chromatography column and washed with buffer containing 0.06% (w/v) digitonin, 200 mM NaCl, 20 mM HEPES (pH 7.5 with NaOH), 2 mM ATP, and 2 mM MgCl_2_. The resin was then incubated for 2 h at 4 °C with 0.35 mg mL^−1^ PreScission Protease to cleave off the GFP tag. The PreScission Protease was removed by dripping eluate through Glutathione Sepharose 4B resin (Cytiva). The protein was then concentrated to yield ~1 mL of sample, which was then phosphorylated by PKA. Finally, protein samples were purified by size-exclusion chromatography at 4 °C using a Superose 6 10/300 GL column (GE Healthcare), equilibrated with a buffer containing 0.03% (w/v) digitonin, 200 mM NaCl, 20 mM HEPES (pH 7.5 with NaOH), 2 mM ATP, and 2 mM MgCl_2_. Peak fractions were pooled and concentrated.

Samples for ATP hydrolysis assays were purified using the same protocol but in a buffer containing KCl, rather than NaCl. The sample used for single-molecule FRET imaging had the following substitutions: C76L, C128S, C225S, C276S, C343S, T388C, C491S, C592M, C647S, C832S, C866S, C1344S, C1355S, C1395S, C1400S, C1410S, S1435C, and C1458S. The purification protocol was adjusted as described in ref. 38. For proteoliposome reconstitution, the size-exculsion chromatography buffer contained glyco-diosgenin (GDN) instead of digitonin.

### EM Data Acquisition and Processing.

Immediately following size-exclusion chromatography, the CFTR (E1371Q) sample was concentrated to 5 mg/mL (32 µM) and incubated with 8 mM ATP, 10 mM MgCl_2_, and 100 µM CFTR_inh_-172 on ice for 30 min. Three mM fluorinated Fos-choline-8 was added to the samples directly before application onto glow-discharged Quantifoil R0.6/1 300 mesh Cu grids. Samples were then vitrified using a Vitrobot Mark IV (Field Electron and Ion Company, FEI).

Cryo-EM images were collected in super-resolution mode on a 300 kV Titan Krios (FEI) equipped with a K3 Summit detector (Gatan) using SerialEM (*SI Appendix*, Table S1). Images were corrected for gain reference and binned by 2. Drift correction was performed using MotionCor2 (57). Contrast transfer function (CTF) estimation was performed using GCTF (58). GCTF values were used for further processing steps. Particles were picked using Gautomatch (https://www.mrc-lmb.cam.ac.uk/kzhang/). All subsequent steps of map reconstruction and resolution estimation were carried out using RELION 3.1 (59) (*SI Appendix*, Fig. S1).

Following the initial round of 3-dimensional classification, the best class was refined and further classified into four classes and processed until no further improvement was observed (*SI Appendix*, Fig. S1*A*). Despite having slightly different nominal resolutions, the top three classes essentially represent the same conformation of CFTR (*SI Appendix*, Fig. S1*B*), with the density for the inhibitor best defined in the highest resolution map (compare *SI Appendix*, Fig. S1*B* with Fig. 2*B*).

### Model Building and Refinement.

Initial protein models were built by fitting the published structure of the NBD-dimerized CFTR (E1371Q) (PDB: 6MSM) into the cryo-EM map using Coot (60). The model was then adjusted based on the cryo-EM density. CFTR_inh_-172 was built into the density and refined in PHENIX (61) using restraints generated by the Global Phasing web server (grade.globalphasing.org). MolProbity (62) was used for geometry validation.

### ATP Hydrolysis Measurements.

Steady-state ATP hydrolysis was measured using an NADH-coupled assay (63). The assay buffer contained 50 mM HEPES (pH 8.0 with KOH), 150 mM KCl, 2 mM MgCl_2_, 2 mM DTT, 0.06% (w/v) digitonin, 60 µg/mL pyruvate kinase (Roche), 32 µg/mL lactate dehydrogenase (Roche), 9 mM phosphoenolpyruvate, 150 µM NADH, and 200 nM CFTR. Aliquots of 27 µL were distributed into a Corning 384-well Black/Clear Flat Bottom Polystyrene NBS Microplate. The reactions were initiated by the addition of ATP. The rate of fluorescence depletion was monitored at λ_ex_ = 340 nm and λ_em_ = 445 nm at 28 °C with an Infinite M1000 microplate reader (Tecan). ATP turnover was then determined with an NADH standard curve.

### Proteoliposome Reconstitution and Planar Bilayer Recording.

The lipids 1,2-dioleoyl-*sn*-glycero-3-phosphoethanolamine (DOPE), 1-palmitoyl-2-oleyl-*sn*-glycero-3-phosphocholine (POPC), and 1-palmitoyl-2-oleoyl-*sn*-glycero-3-phospho-L-serine (POPS) were mixed at a 2:1:1 (w/w/w) ratio and resuspended by sonication in buffer containing 200 mM NaCl, 20 mM HEPES (pH 7.2 with NaOH) and 2 mM MgCl_2_ to a final lipid concentration of 20 mg/mL. Then, 2% (w/v) GDN was added and the mixture was incubated for 1 h at 25 °C. CFTR was mixed with the lipids at a protein-to-lipid ratio of 1:250 (w/w) and incubated at 4 °C for 2 h. Following that, 14 mg/mL methylated beta-cyclodextrin was added to the mixture. After 4 h, an equivalent amount of methylated beta-cyclodextrin was added to the mixture. This was performed for a total of four additions. Proteoliposomes were pelleted by centrifugation at 150,000 g for 45 min at 4 °C and resuspended in buffer containing 200 mM NaCl, 20 mM HEPES (pH 7.2 with NaOH), and 2 mM MgCl_2_.

Synthetic planar lipid bilayers were made from a lipid mixture containing DOPE, POPC, and POPS at a 2:1:1 (w/w/w) ratio. Proteoliposomes containing PKA-phosphorylated CFTR were fused with the bilayers. Currents were recorded at 25 °C in a symmetric buffer containing 150 mM NaCl, 2 mM MgCl_2_, 20 mM HEPES (pH 7.2 with NaOH), and 3 mM ATP. Voltage was clamped at −150 mV with an Axopatch 200B amplifier (Molecular Devices). Currents were low-pass filtered at 1 kHz, digitized at 20 kHz with a Digidata 1440A digitizer and recorded using the pCLAMP software suite (Molecular devices). Recordings were further low-pass filtered at 100 Hz. Data were analyzed with Clampfit, GraphPad Prism, and OriginPro. A lower bound of 10 ms was put on event duration in idealization of channel recordings to exclude flicker-closures from the dwell time analysis. Reported open dwell times thus reflect open burst dwell times.

### Single-Molecule Fluorescence Imaging and FRET Data Analysis.

Imaging and analysis were carried out as outlined in refs. 38 and 64. In brief, PKA-phosphorylated CFTR was immobilized within PEG-passivated microfluidic chambers via a streptavidin–biotin–tris-(NTA-Ni^2+^) bridge. Experiments were performed at 25 °C in buffer containing 0.06% (w/v) digitonin, 150 mM NaCl, 2 mM MgCl_2_, 20 mM HEPES (pH 7.2 with NaOH), 2 mM protocatechuic acid, and 50 nM protocatechuate-3,4-dioxygenase.

Imaging was carried out with a custom-built wide-field, prism-based total internal reflection fluorescence microscope. Donor (LD555) fluorophores were excited with an evanescent wave generated using a 532-nm laser (Opus, Laser Quantum). Fluorescence emitted from donor (LD555) and acceptor (LD655) fluorophores was collected with a 1.27 NA 60× water-immersion objective (Nikon), spectrally resolved using a T635lpxr dichroic (Chroma), and imaged onto two Fusion sCMOS cameras (Hamamatsu). The integration period of imaging was 100 ms.

Analysis of fluorescence data was performed using the SPARTAN analysis software in MATLAB (65). Single-molecule FRET trajectories were calculated as *E_FRET_* = *I_A_*/(*I_A_* + *I_D_*), where *I_A_* and *I_D_* are the emitted acceptor and donor fluorescence intensities, respectively. The following pre-established criteria were applied to select FRET trajectories for analysis: Single-step donor photobleaching; a signal-to-noise ratio above 8; fewer than 4 donor-blinking events; FRET efficiency below 0.8; and FRET efficiency above baseline for at least 50 frames. The segmental k-means algorithm (66) was used to idealize trajectories to a model containing two non-zero-FRET states with FRET efficiencies of 0.25 and 0.48. Data were further analyzed with OriginPro. Probabilities of high FRET occupancy are likely slightly underestimated as a small fraction of molecules were nonresponsive to ATP exposure. This population likely reflects denatured molecules. These molecules cannot be excluded in an unbiased manner based on their photophysical properties and are therefore included in our analysis.

## Supplementary Material

Appendix 01 (PDF)

## Data Availability

The cryo-EM map of CFTR_inh_-172-bound CFTR has been deposited in the Electron Microscopy Data Bank under the accession code EMD-42101 (67). The corresponding atomic model has been deposited in the Protein Data Bank under accession code 8UBR (68). All other data and information are available in the main text or *SI Appendix*.
