# Supplementary material for: Structural basis for CFTR inhibition by CFTRinh-172
Source: Proc Natl Acad Sci U S A. 2024 Feb 29;121(10):e2316675121. doi: 10.1073/pnas.2316675121 (PMC10927578; doi:10.1073/pnas.2316675121)
Supplement: Supplementary file 1 — Appendix 01 (PDF) [file pnas.2316675121.sapp.pdf]

Table S1. Cryo-EM data collection, refinement, and validation statistics

	CFTR (E1371Q)/CFTR$_{inh}$-172 +ATP/Mg^{2+} (EMDB-42101) (PDB 8UBR)
**Data collection and processing**	
Magnification	105,000
Voltage (kV)	300
Electron exposure (e^-/Å^2)	65.6
Defocus range (μm)	0.5-2.5
Pixel size (Å)	0.676
Symmetry imposed	C1
Initial particle images (no.)	782,712
Final particle images (no.)	66,267
Map resolution (Å)	2.70
FSC threshold	0.143
Map resolution range (Å)	2.7-3.8
**Refinement**	
Initial model used (PDB code)	6MSM
Model resolution (Å)	2.6
FSC threshold	0.143
Map sharpening *B* factor (Å^2)	-43.286
Model composition	
Non-hydrogen atoms	9440
Protein residues	1156
Ligands	9
B factors (Å^2)	
Protein	98
Ligand (inhibitor)	67
R.m.s. deviations	
Bond lengths (Å)	0.070
Bond angles (º)	0.870
Validation	
MolProbity score	1.17
Clashscore	3.811
Poor rotamers (%)	0.1
Ramachandran plot	
Favored (%)	98.25
Allowed (%)	1.75
Disallowed (%)	0

Table S2. **List of oligonucleotides used in this study**

**Oligonucleotides used for mutagenesis**	**Source**
Primer: K95A forward:<br>GCGAGGTTACTGCTGCCGTACAGCC	IDT
Primer: K95A reverse:<br>GGCTGTACGGCAGCAGTAACCTCGC	IDT
Primer: S1141A forward:<br>CAATGAACATCATGGCTACACTTCAATGGGCAG	IDT
Primer: S1141A reverse:<br>CTGCCCATTGAAGTGTAGCCATGATGTTCATTG	IDT
Primer: T1142I forward:<br>CAATGAACATCATGTCTATTCTTCAATGGGCAGTTAAC	IDT
Primer: T1142I reverse:<br>GTTAACTGCCCATTGAAGAATAGACATGATGTTCATTG	IDT
Primer: T1142A forward:<br>GGCAATGAACATCATGTCTGCACTTCAATGGG	IDT
Primer: T1142A reverse:<br>CCCATTGAAGTGCAGACATGATGTTCATTGCC	IDT
Primer: E1371Q forward:<br>ACCTTCTGCTCACTTGGACCCCGTTACATACCAAATC	IDT
Primer: E1371Q reverse:<br>GGTCCAAGTGAGCAGAAGGTTGATCAAGCAGCAAGATCTTGGC	IDT

Figure S1

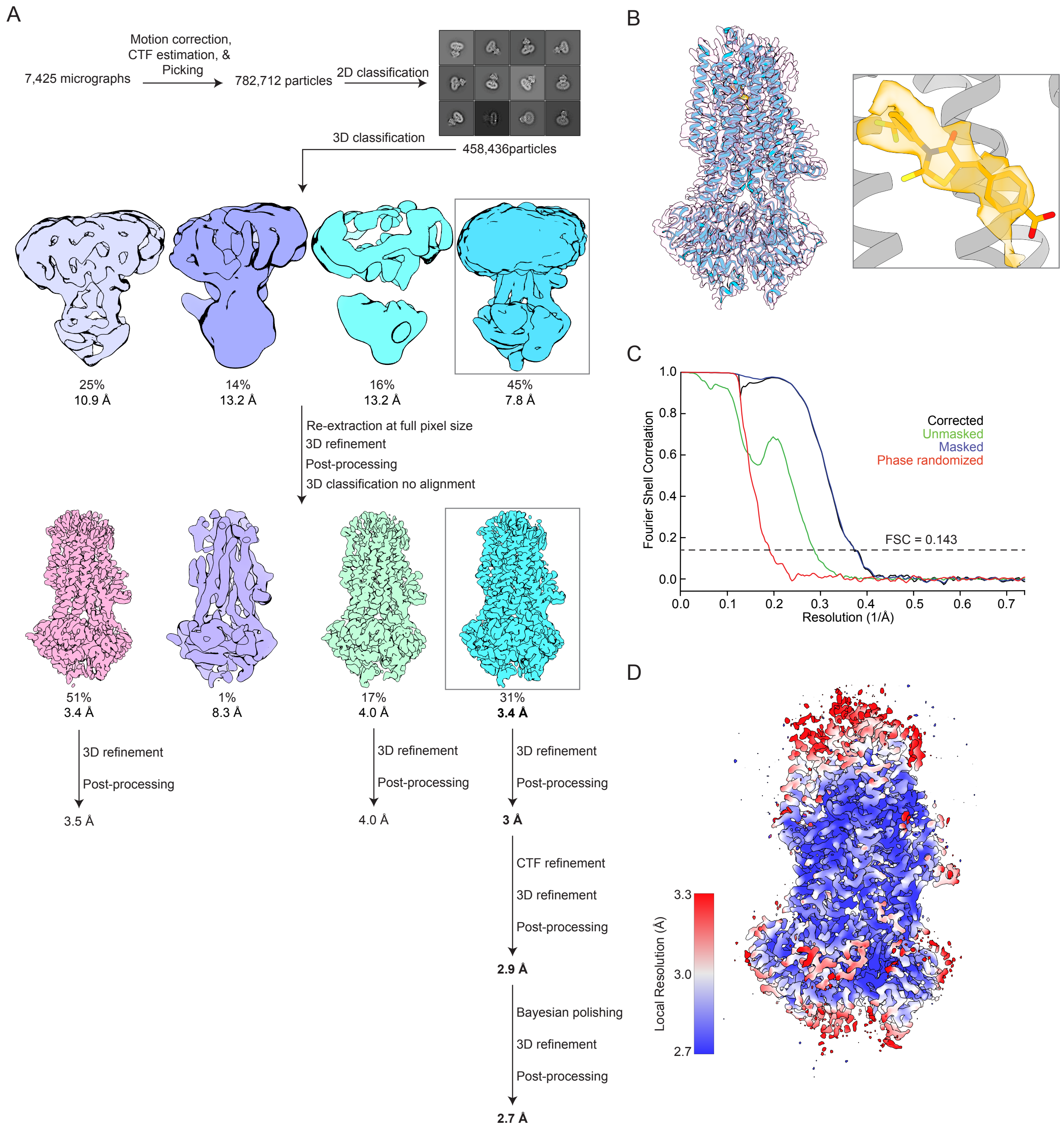

Figure S1. Cryo-EM analysis of the CFTR/CFTR$_{inh}$-172 complex and quality of the reconstruction. (**A**) Image processing procedure. (**B**) (left) Superposition of final model (blue) with the 51%-occupancy class (shown as pink surface) from the second round of 3D classification. (right) CFTR$_{inh}$-172 modeled into its density (shown as orange surface) in the 51%-occupancy class from the second round of 3D classification. (**C**) Fourier shell correlation curves of the final map. (**D**) Local resolution estimation of the final map.

Figure S2

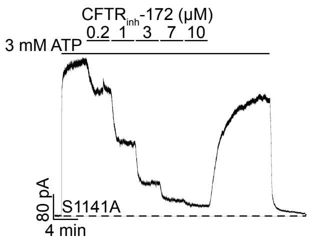

Figure S2. CFTR$_{inh}$-172 titration onto S1141A CFTR. Example macroscopic current traces showing titration of CFTR$_{inh}$-172 onto S1141A CFTR in inside-out excised patches. CFTR was fully phosphorylated by PKA in the presence of 3 mM ATP before CFTR$_{inh}$-172 titration.
